# Lactoferrin and Its Detection Methods: A Review

**DOI:** 10.3390/nu13082492

**Published:** 2021-07-22

**Authors:** Yingqi Zhang, Chao Lu, Jin Zhang

**Affiliations:** Department of Chemical and Biochemical Engineering, University of Western Ontario, London, ON N6A 5B9, Canada; yzha2268@uwo.ca (Y.Z.); clu248@uwo.ca (C.L.)

**Keywords:** lactoferrin, biomarkers, immunoassay, instrumental analysis, sensor

## Abstract

Lactoferrin (LF) is one of the major functional proteins in maintaining human health due to its antioxidant, antibacterial, antiviral, and anti-inflammatory activities. Abnormal levels of LF in the human body are related to some serious diseases, such as inflammatory bowel disease, Alzheimer’s disease and dry eye disease. Recent studies indicate that LF can be used as a biomarker for diagnosis of these diseases. Many methods have been developed to detect the level of LF. In this review, the biofunctions of LF and its potential to work as a biomarker are introduced. In addition, the current methods of detecting lactoferrin have been presented and discussed. We hope that this review will inspire efforts in the development of new sensing systems for LF detection.

## 1. Introduction

Lactoferrin (known as lactotransferrin, LF), with a molecular weight of about 80 kDa, is a functional glycoprotein, which contains about 690 amino acid residues. It was first isolated from bovine milk by Sorensen in 1939 and was first isolated from human milk by Johanson in 1960 [1,2]. The three-dimensional structure of LF has been unveiled by high resolution X-ray crystallographic analysis, and it consists of two homologous globular lobes with four domains [3]. The high level of flexibility of LF structure is related to various bio-functions in the human body, such as host defense, inhibition of tumor growth, enzymatic activity of ribonuclease A, antimicrobial activity, cell proliferation and differentiation regulation, antibacterial activity, antiviral activity and antiparasitic activity [4].

As a member of the transferrin family, LF is also considered an iron-binding glycoprotein because of its ability to bind Fe^3+^ ions [5]. Pioneering work has demonstrated that LF has high affinity for ferric iron (with K_D_ around 10^−20^ M [6]) and plays the predominant role in regulating free iron level in the body fluids [7,8,9]. Although LF has many similarities with other transferrins (TF), differences of localization of glycosylation sites have been observed between LF and serum TF. The asparagine residues 137 and 490 of LF are glycosylated while serum TF has glycosylated residues on Asn-Lys-Ser (residues 428–430) and Asn-Val-Thr(residues 635–637) [10,11]. In addition, human transferrin has disulfide bonds at the two cysteine residues (amino acids 331 and 339), while there are no such bonds on LF [10,12,13]. Besides, the mechanisms for transporting iron of LF and TF are different. The human milk LF has a much higher iron binding equilibrium [14] than serum TF and is able to retain iron under much lower pH (around 3.0) than its counterparts in the transferrin family. This may be attributed to the cooperative interactions between the N-lobes and C-lobes in the molecule structure [6]. When binding to the iron, the C-lobe of LF has a higher rotation degree than that of human transferrin, because of the helical inter-lobe linker in LF.

The difference in iron saturation of LF may have an effect on its biofunctions. For example, LF with higher iron content could improve antimicrobial activity via inhibiting the growth of bacteria [15], increasing the cell membrane permeability [16] or generating LF hydrolysate [17], while LF with a lower iron-saturation degree would provide more capacity for binding iron to decrease antioxidant ability [18]. Since LF has a characteristic absorption at around 465 nm, the simplest method to determine the degree of iron saturation is to use UV–visible spectrophotometry. However, this method can be applied for determining samples which only contain apo-LF or holo-LF [19,20,21]. However, for complicated samples, inductively coupled plasma–mass spectrometry (ICP-MS) can be considered as a way of obtaining higher accuracy [21,22,23]. In addition, atomic absorption spectrometry can also be applied for such determination [24,25].

To better understand the role of LF in maintaining human health, many efforts have been made for several decades. The major functions of LF related to the antioxidant, antibacterial, antiviral, anti-inflammatory activities have been investigated. Recent studies indicate that LF can be considered as a biomarker in the diagnosis of some diseases, such as inflammatory bowel disease (IBD), Alzheimer’s disease (AD) and dry eye disease (DED) [26,27,28,29,30,31,32,33]. Instrumental analysis (high performance liquid chromatography and capillary electrophoresis), immunoassay method (radial immunodiffusion and enzyme-linked immunosorbent assay) and various sensors (fluorescence, electrochemical and surface plasmon resonance) have been studied for the measurement of LF, but none of them are satisfactory. Consequently, there is a need for developing a detection method for rapid and accurate measurement of LF.

In this review, we briefly introduced various biofunctions of LF and its potential role as a biomarker for the diagnosis and management of different diseases. This review highlights the analytical strategies for measuring the concentration of LF. Meanwhile, it gives a comprehensive comparison of different kinds of methods.

## 2. Bio-Functions of Lactoferrin

### 2.1. Antioxidant Activity

Many studies on the antioxidant activity of LF have been conducted in vivo. Rats were normally used in animal tests. The chronic administration of LF would significantly reduce the elevated plasma H_2_O_2_ and production of reactive oxygen species [34,35].

### 2.2. Anti-Inflammatory Activity

LF plays an important role in immune defense, such as the genital, gastric and ophthalmic mucosal defense systems. When responding to inflammatory stimuli, the expression of LF would be upregulated in those sites to inhibit the production of inflammatory cytokine and the binding ability of lipopolysaccharide endotoxin to inflammatory cells [5,36]. In addition to inducing systemic immunity, it was also proved to be capable of inhibiting allergens and lowering the severity of local cutaneous inflammatory reactions [37].

### 2.3. Antibacterial Activity

It has been proved that LF is able to inhibit the growth of various bacterial pathogens [38], such as *S. mutans*, *S. epidermidis*, *E. coli* and so on. Several mechanisms have been speculated to explain the bactericidal effects of LF. Arnold et al. have proved that its binding ability to iron would impede iron utilization by bacteria and inhibit their growth [15]. Moreover, the death of bacteria cells can be induced by the disruption of cell walls, which was caused by the interaction between the N-terminal region of LF and related receptors, e.g., lactoferrin binding protein A and/or B on Gram-negative bacteria [16] or electrostatic interactions with Gram-positive bacteria [39]. LF was also proved to have innate antibacterial properties via its hydrolysate, an antimicrobial peptide, which makes the colonies hard to form [40].

### 2.4. Antiviral Activity

In addition to the antibacterial activity, many studies have demonstrated that LF also exhibits antiviral activity on both DNA- and RNA-viruses, including herpesvirus [41], human immunodeficiency virus (HIV) [25] and rotavirus [42]. This antiviral effect is proved to be achieved by LF’s ability to block cellular receptors or binding to the virus particles [43,44].

### 2.5. Anti-Tumor Activity

Previous studies have demonstrated the inhibition effect of LF on the growth of tumor cells via direct cellular inhibition and/or systemic immunomodulation [45,46,47]. It shows that LF has a dose-dependent anti-cancer efficiency in the treatment of lung cancer [48]. In addition, LF also has a potent synergistic effect in chemotherapy on the production of cytokines in tumor cells [47].

### 2.6. Activity as a Growth Factor

The potential biofunction of LF as a growth factor has been studied on various cell lines. Rather than directly supporting the growth and proliferation of cells, its ability as an activator of growth factor and its synergistic effect with other growth factors for growth-stimulating has been observed in vitro by using rat intestinal epithelial cell line [49] and human lymphocytic cell line [50]. The direct proliferation effect on bone cells, and promoting effect on alkaline phosphatase activity and calcium deposition were observed and confirmed recently in the rat osteoblast cell line [51]. A hydrolyzed peptide from LF was found to interact with a key domain of epidermal growth factor receptor by interpolated charge, hydrophobicity, and hydrogen bonding.

## 3. Lactoferrin as Biomarker

To achieve early-stage diagnosis and personal disease management, it is vital to use suitable biomarkers, which are “an indicator of normal biological processes, pathogenic processes or responses to an exposure or intervention” [52]. They can be categorized into diagnostic, monitoring, pharmacodynamic, prognostic and predictive biomarkers [53,54]. They can provide a powerful tool to understand the prediction, cause, diagnosis, progression, regression, or outcome of treatment of disease, such as glucosyl sphingosine, a biomarker for diagnosis of Gaucher disease [55], subregional neuroanatomical for Alzheimer’s disease [56], and serum CA 19-9 for pancreatic cancer [57]. LF can be found in fecal, milk, serum, tears and other secretions from human body, and has been reported as a biomarker to indicate several diseases, such as inflammatory bowel disease (IBD) [26,27], Alzheimer’s disease (AD) [28] and dry eye disease (DED) [29,30,31,32,33].

### 3.1. Inflammatory Bowel Disease (IBD)

Based on a systematic review, the incidence and prevalence of IBD increased recently, especially in Asia [58,59]. Traditionally, the determination of IBD has mostly been relied upon in clinical scoring systems and endoscopy, which are expensive and have low accuracy [60,61]. Some previous studies indicated that fecal LF has the potential to act as the biomarker for IBD, both Crohn’s disease (CD) and ulcerative colitis (UC), but its performance in diagnosing UC patients was better than that in CD patients [62,63,64]. During intestinal inflammation, the secondary granules are released as polymorphonuclear neutrophils degranulate [65,66]. Since the major component of secondary granules is LF, which has antibacterial and anti-inflammatory properties [11,67], an increased LF concentration can be observed in IBD [68]. Thus, LF may be considered as a good biomarker to predict IBD [69] both in patients with UC and CD [70].

Buderus’ team believed that fecal LF is a reliable biomarker for active inflammatory bowel disease (IBD) in pediatric patients. It was found that the levels of fecal LF of both CD and UC patients were higher than that of control subjects (<7.3 μg/g) [62]. Prata’s research compared the concentrations of LF in frozen fecal specimens from 78 children in Brazil by using ELISA, which also indicated that LF can be considered as a biomarker of intestinal inflammation [71]. The studies of Kane’s group and Dai’s group showed that fecal LF can be used as a biomarker for the diagnosis of IBD. They studied the level of fecal LF of IBD (CD and UC) patients, irritable bowel syndrome (IBS) patients, and healthy controls; the results indicated that the concentration of fecal LF of IBD patients was significantly higher than that of IBS patients and controls [26,27]. Wang’s research team conducted a systematic review with a meta-analysis by using the Medline and EMBASE databases, and confirmed that fecal LF can be used for accurate diagnosing of IBD. Besides, specificity of fecal LF for IBD diagnosis is 100%, and the sensitivity for CD diagnosis is 75% and for UC diagnosis is 82% [64].

### 3.2. Alzheimer’s Disease (AD)

It is a challenge to have an early-stage diagnosis of Alzheimer’s disease (AD). Current strategies lie in the evaluation of the levels of cerebrospinal fluid (CSF) tau and amyloid β (Aβ) by integrating the techniques of positron emission tomography (PET) and magnetic resonance imaging (MRI) [72,73,74,75]. Efforts have been made to develop a quick and cost-effective method for the diagnosis of AD. Accumulated evidence indicated that bacterial and viral infections may cause AD [76,77,78] and lead to a deteriorated innate immune system in AD pathophysiology [28]. Since saliva with many antimicrobial proteins is considered as the first line of the body’s defense [79], there are some reports on the relationship between oral infections and AD [80,81]. In saliva, LF acts as one of the most important defensive elements due to its unique antimicrobial activities [82]. Therefore, salivary LF level could be considered as a promising biomarker to aid the diagnosis of AD at an early stage.

Contrary to the upregulation of LF in brain tissue, the work done by Carro’s group [79] observed decreased LF concentration in unstimulated saliva from AD patients by comparing to the control, and the results were more accurate than those obtained from analyzing biomarkers such as total tau and CSF Aβ_42_ in cerebrospinal fluid. Besides, this study also proved that apparently healthy participants but with low levels of salivary LF would have a relative high possibility of AD in the future. González’s study continued to use salivary LF to diagnose prodromal AD and further studied the relationship between salivary LF and cerebral amyloid-β (Aβ); the result showed that salivary LF levels would not decrease in other dementias, such as the frontotemporal dementia, and reduced LF may be attributed to the disruption of hypothalamic function because of the early hypothalamic Aβ accumulation [28].

### 3.3. Dry Eye Disease (DED)

Dry eye disease, a common ocular surface disease of multifactorial etiology, may cause plenty of symptoms and visual impairment, potentially with ocular surface damage [83,84]. DED can currently be diagnosed by evaluating the tear osmolarity, Schirmer tear test, phenol red thread test, etc. [85,86]. However, these methods tend to have low accuracy and can be easily affected by environmental factors. Lactoferrin plays a key role in the tear film to avoid ocular diseases because of its unique biofunctions (antimicrobial and anti-inflammatory activities) [87]. LF can scavenge oxygen free radicals and hydroxyl in normal tears, but these activities are inactive in DED tears due to the level of LF. The reduction of it will expose eyes to additional oxidative metabolites which may cause higher susceptibility [88,89]. Besides, some recent researches have confirmed that the concentration of LF in tears is significantly different between patients with dry eye disease and controls [29,30,31,32,33]. The drop of quality or quantity of the tear film are main abnormalities of DED [90]. It was also reported that LF is one of the important predictors of the stability and/or volume of tear film. Tear volumes from the lacrimal gland are observed to have a positive correlation with the concentration of LF. Patients with lower tear production tend to have lower LF concentration [91,92]. The level of LF in tears of DED has great potential to be considered as a novel biomarker for determining DED [29,30,31,32,33].

Seal’s research results from detecting concentrations of various proteins in tears by using ELISA indicated that tear LF concentrations in normal people were more than two standard deviations higher than that in the sicca patients [30]. Boukes’s team collected human tears with Schirmer strips and analyzed them by using HPLC to detect tear protein profiles in patients with dry eye. Followed by comparing with those in a control, it was found that the concentrations of tear LF in the control were around five times higher than those in patients, especially in the age group 40–50 years [31]. Versura’s research group focused on studying levels of various proteins in tears of patients with evaporative dry eye (tear film break-up time ≤ 10 s) disease and compared the results with healthy subjects (tear film break-up time ≥ 10 s). They separated tear proteins by SDS-PAGE electrophoresis and identified them by mass spectrometer and Western blot analysis. It was indicated that levels of LF statistically significantly decreased in evaporative dry eye patients [33].

## 4. Analytical Strategies for Lactoferrin

As LF from different secretions of the human body has been reported as a biomarker for different diseases in recent decades, investigation into developing an accurate, cheap and fast way has attracted more attention, which could help diagnose diseases at an early stage. Various detection methods have been studied and have proved their ability in the quantification of LF with high accuracy and sensitivity. Immunoassay, instrumental analysis, fluorescence-based biosensors, electrochemical-based biosensors/sensor, and surface plasmon resonance (SPR) sensors will be discussed and compared in this section.

### 4.1. Immunoassay

#### 4.1.1. Radial Immunodiffusion (RID)

**Figure 1:**
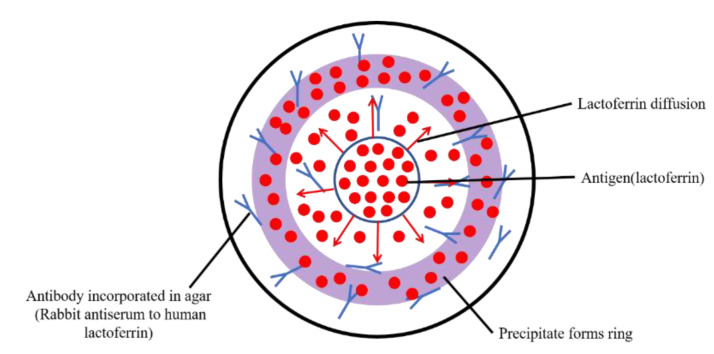
Scheme of radial immunodiffusion. Lactoferrin from the center would conjugate with its antibody and diffuse along the agar, and the area of the ring reflects its concentration.

Single radial immunodiffusion is a relatively simple quantitative approach for antigen without using expensive and integrated instruments, and has been developed based on the immunochemical precipitin method by applying the diffusion of antigen in antibody-conjugated agar gels. The antigen is allowed to diffuse radially through a uniform thin-layer of antibody-containing agar and form a circle of precipitin. The final area can be used to indicate the concentration of the antigen (as is shown in Figure 1).

Janssen’s study on detecting concentrations of tear LF was carried out by developing a radial immunodiffusion assay, which was applied with rabbit antiserum to human LF as antibody in agar gels [93]. Purified LF solutions with the concentration of 0.25–4 mg/mL were used as standard samples, and the circles formed by tear samples were compared with the standard rings for the estimation of concentration. Although this method is easy to operate and the sample volume used for detection is small, various impact factors may limit the detection range and impair the detection accuracy. The detection range is largely dependent on the standard samples and the introduction of error is unavoidable because the depth and density of agar plate cannot be guaranteed to be absolutely uniform at any site, which makes the diffusion of antigen heterogeneous, and the error on the measurement of the area of precipitin circles still exists. Besides, each plate is required to have individual standard precipitin rings, and the immunochemical precipitin method was considered as time consuming, expensive, and lacking accuracy.

#### 4.1.2. Enzyme-Linked Immunosorbent Assay (ELISA)

**Figure 2:**
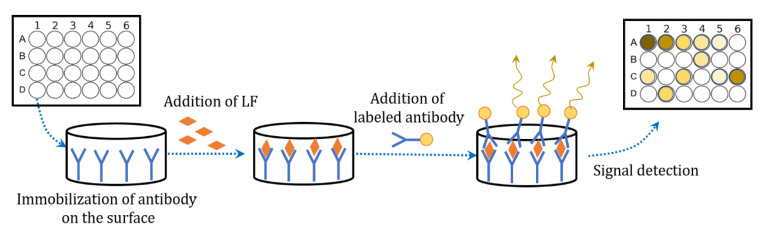
Scheme of sandwich ELISA method for lactoferrin detection.

Enzyme-linked immunosorbent assay (ELISA) is a successful, rapid and accurate immunological analysis technique based on the specific reactions of antigens and antibodies, and has been widely applied on the quantification of proteins with high selectivity and accuracy (Figure 2). Many articles reported the application of ELISA on LF detection. It was reported that a sandwich ELISA method by applying rabbit anti-LF to the assay was able to measure LF in cows’ milk, cheeses and their whey with a detection limitation of 18 ng/mL [94]. Liu’s group also tried to detect the concentration of LF in milk powder by using sandwich ELISA assay. They prepared monoclonal antibodies based on hybridoma techniques and then labeled them with horseradish peroxidase for utilizing as a detection antibody. This kind of assay obtained the limit of detection (LOD) of 3.23 ng/mL with a linear detection range of 5–600 ng/mL [95].

ELISA can detect dozens of samples of LF in low concentration and the states of samples can be various, such as serum [96,97], saliva [98,99], tears [100] and milk products. It is now considered as an ideal method for the detection of LF. However, the price of the reagent kits is relatively expensive and the process of doing ELISA is laborious and time consuming.

### 4.2. Instrumental Analysis

LF, with the specific ultraviolet absorbance, can feasibly be directly qualified and quantified by UV-Vis spectroscopy (at 280 nm [101] or 220 nm [102]). However, such a direct detection method is only applied for the pure and simple sample. Poor signal-to-noise ratio is normally observed in an impure sample. Therefore, the separation processes before the spectroscopy detection are essential. Meanwhile, the separation efficiency also significantly affects the limitation and accuracy of these methods. Electrophoresis separation and column separation are considered effective and are widely used for purifying biomolecules; previous researches also proved their potential application in the separation of LF.

#### 4.2.1. Reversed Phase-High Performance Liquid Chromatography (RP-HPLC)

HPLC has become vital technology used for separation and characterization of proteins and peptides. Based on the differences of polarity and non-polarity in the stationary phase and the mobile phase, HPLC can be divided into normal HPLC and RP-HPLC. After the sample mixture is introduced into the mobile phase and goes through the column, the components will be separated by different retention times due to their various structures and properties, and the detector at the end is used for quantifying. In the detection of LF by chromatography, RP-HPLC has been widely applied and studied. C18 or C4 reverse phase columns are mostly used as the stationary phase, while aqueous solution is employed as the mobile phase. The UV detector at the end showed great sensitivity and good baseline at 220 nm for LF [103].

The work done by Palmano’s group demonstrated linear calibration for the quantitative measurement of LF by using RP-HPLC, in which C4 column and sodium phosphate buffer were used as stationary and mobile phase, respectively. However, the result of this method was largely affected by other proteins, such as bovine serum albumin (BSA), and the peak of LF could not be distinguished if the ratio of BSA to LF exceeded 10:1 [104]. Wen et al. improved the existing method by choosing C18 column and using a mixture of water and acetonitrile as the mobile phase, and further studied the LF content in simulated gastrointestinal fluids. The LOD was significantly lowered to 1 μg/mLand the result could be acquired within 20 min, which exhibited its feasibility in biomedical application [105]. However, the samples in this article are stimulated and standard samples, in which no other interfered proteins existed. After that, a two-step RP-HPLC quantification method was developed to increase the selectivity of LF. The instrumental condition was similar to that in Wen’s method, but the column was changed to C4 column. Before entering the column, the samples were absorbed and desorbed on the resin. In this process, proteins with higher isoelectric points (pI) such as lactoferrin can be selectively adsorbed on cation-exchange materials, followed by desorption. The recovery rate of resin is high (up to 98%) and linear relationship of LF from 25 to 514 μg/mL was obtained. However, the purification process was time-consuming and would bring much more error on quantification. C18 and C4 columns both showed sensitivity, accuracy and reproducibility. Comparing to the C18 column, the lower C-loaded column showed narrower and better shape peaks for LF and was easier to clean [102,103]. Although RP-HPLC is regarded as a time-saving method for detection with high accuracy, sensitivity and selectivity, some proteins which have pI with LF tend to have similar retention time and make it difficult for the target protein to be distinguished by the existing column.

In addition, the physicochemical properties of LF with different iron contents (apo-, native- and holo-LF) are various, which may affect the separation performance of the column [106,107]. To avoid such influence, a tryptic signature peptide, which is a hydrolysate from bovine LF, was chosen and identified as the representative of LF based on a sequence database search. Pretreatment of samples containing LF, involved in enzymatic digestion, centrifugation and purification, is essential in this method. After the separation by ultra-HPLC, the analyte was measured and quantified by electrospray ion source of a mass spectrometer (ESI-MS) detector [106]. This method showed good linear relationship between signal intensity and concentration of LF, and increased the precision of chromatography to the level of nmol/L.

#### 4.2.2. Capillary Electrophoresis

**Figure 3:**
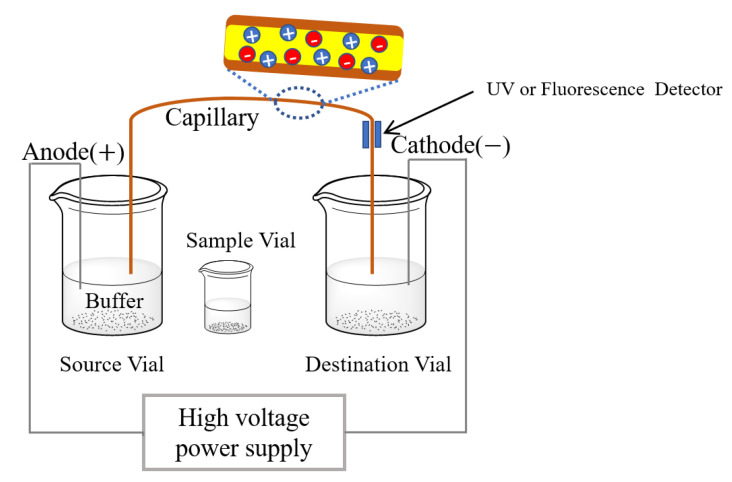
Mechanism of detecting lactoferrin by capillary electrophoresis (CE).

Capillary electrophoresis (CE) is a family of electrophoresis method, which is a traditional way of using the electrical field to separate and purify charged biomolecules. The capillaries or micro/nano-fluid tubes are used as separation channels, which increased the surface to volume ratio, separating efficiency and capabilities. The structure of CE instrument is relatively simple, as shown in Figure 3. An electrical field is firstly formed between the source and destination vials and inside a capillary by the high-voltage power supply. Then, samples are introduced into the capillary by placing capillary inlet into the sample vials. All negatively or positively charged molecules would be separated under a high voltage power because of their different electrophoretic mobilities. Finally, the separated molecules are analyzed by UV or fluorescence detector [108].

However, it is still hard to directly use CE for the detection of LF in whey samples because LF may be adsorbed on the capillary wall, which results in poor separation and low accuracy. Thus, fluorescein isothiocyanate-conjugated polyanionic lipopolysaccharide was added for minimizing the interaction between the positively charged LF and the capillary wall, which may result in a significant migration time shift on the electropherogram. Thus, the intensity of fluorescence could reflect the concentration of LF [109]. It is noted that this method is only applicable for the sample with high LF concentration (tens of micrograms per milliliter). However, recent progress on improving CE method for detecting LF has been recognized by sample pretreatment, by adjusting buffer solution and surface modification of capillary wall to reduce the affinity between LF and column wall. 

A method to determine levels of LF in infant formula was reported by utilizing the uncoated capillary for separation in the optimized buffer solution followed by UV at 214 nm for detecting. Samples need to be pretreated by acetic acid, and non-ionic surfactant is added into the buffer solution to suppress the LF adsorption on the capillary wall. The results showed a linear relationship between the peak areas and the concentrations of LF with the limit of detection at 3 mg/L and the limit of quantitation at 10 mg/L [110]. Different from the previous work, Mao’s group developed a method to measure the concentration of LF in infant formula by using the capillary coated with poly(2-methyl-2-oxazoline)-random-glycidyl methacrylate copolymer rather than pretreat samples. This kind of modified column exhibited high separation efficiency for basic proteins, and a good linear relationship between 10–500 μg/mL was achieved with the LOD of 5 μg/mL and the limit of quantitation of 16.7 μg/mL [111].

The selectivity of CE method is largely dependent on the efficiency of separation. The proteins with similar properties and electrophoretic mobility in complex samples may become hard to distinguish and result in large error in the LF detection. Furthermore, CE-based systematic evolution of ligands by exponential enrichment (CE-SELEX) was applied and a more accurate result with selectivity and lower LOD could be obtained [112]. This technology was firstly used to screen the ssDNA aptamer with high affinity for LF. Followed by the selection process, such aptamer was mixed with LF before being injected into the capillary. Since the binding affinity between LF and aptamer exists, the aptamer-conjugated LF could be easily separated and distinguished by CE, and the result showed good linear relationship on 4–128 nM LF with LOD of 1 nM (around 78 ng/mL). The samples discussed here are mainly whey samples or milk powder and the application of CE on a biological sample has not been reported so far. Although the application of CE on LF detection is mainly used in food science, this method still exhibits excellent potential in the analysis of body fluids (plasma, tear, saliva and cerebrospinal fluid).

### 4.3. Other Sensors

Microfluid device and nano-based electrochemical or colorimetric sensors are platforms which could detect LF in an accurate and real time way. These devices are typically lightweight and do not require advanced instruments, which makes them have more application scenarios.

#### 4.3.1. Fluorescence-Based Biosensor

**Figure 4:**
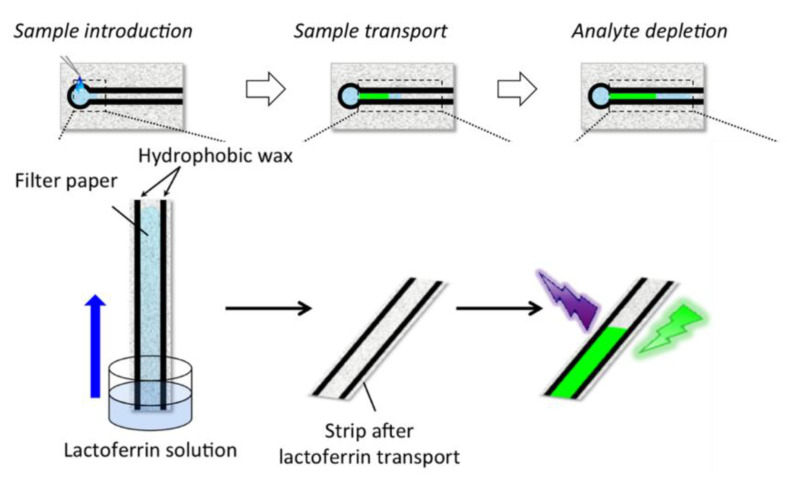
Scheme of detecting process of distance-based microfluidic paper device (reprinted with permission) [114].

A novel microfluidic paper-based analytical device (μPAD) to measure the concentration of LF was reported by Kudo with a limit of detection of 110 μg/mL [113]. Such devices tend to have three main processes during the detection (Figure 4; namely the introduction of samples, followed by sample transportation and detection). This method utilized the high affinity between LF and ferric ions, and the color change which was caused by the replacement of the indicator from the complex of a colorimetric 2-(5-bromo-2-pyridylazo)-5-diethylaminophenol (5-Br-PADAP)-Fe^3+^ by LF, was considered as a signal for detection. The distance of the color changed area from the origin reflected the concentration of LF in the solution. Only a color readout app is needed to distinguish the boundary of different colors on the paper, and such paper-based device with good accuracy are easy to apply without any other instrument. However, the sample volume used for detection was 40 μL, which is relatively larger than other methods and not suitable for small sample analysis. Besides, the nonuniformity of papers’ cellulose substrate may affect the precision of colorimetric line and should be considered in this paper device. Further, Yamada’s research group developed another kind of distance-based μPAD for detecting LF by applying the fluorescence emission properties of the conjugation of LF and trivalent terbium as a signal. By analyzing the length of fluorescence on the μPAD channel, this method could reach a limit of detection of 0.1 mg/mL [114]. The dimension of the channel in the microfluid device was carefully modified and the sample volume was largely reduced to 2 μL. The paper was also treated on both sides by wax printer and anionic polysaccharides to eliminate the factor of heterogeneity of fiber.

**Figure 5:**
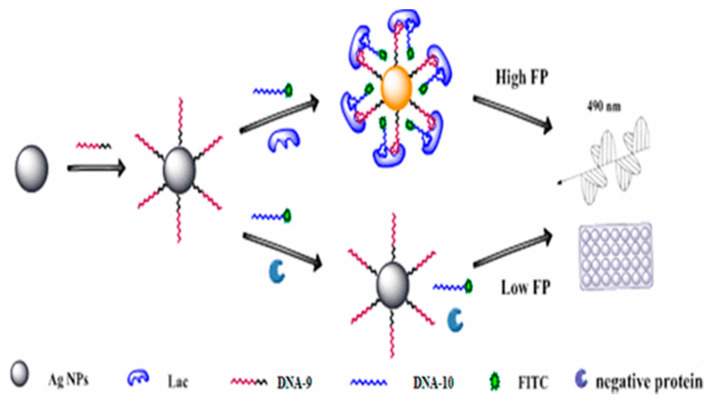
Scheme of bivalent aptasensor for detecting lactoferrin by using fluorescence polarization (reprinted with permission) [115].

In addition to the μPAD, fluorescence-labeled bivalent aptamer-sensor was developed for the detection of LF [115] with the detection limit of 1.25 pM. Such a sensor took advantage of more than one amplification strategy to achieve sensitivity, selectivity and high amplification factor. Two different aptamers (DNA-9 and DNA-10) with high affinity for LF were screened by SELEX. DNA-10 was conjugated with fluorescein isothiocyanate (FITC), which was used for the generation of fluorescence after linking to the LF, and the DNA-9 was immobilized on the surface of silver nanoparticles (Ag NPs), which could enhance the intensity of the signal by the mass-augmented and metal-enhanced fluorescence (MEF) effect (Figure 5). This biosensor has good linear relationship between 0.2 ng/mL and 25 μg/mL, the range of which is relatively wide, and the determination limit (0.2 ng/mL) was much lower than that of the current methods, even than ELISA. The good recovery rates of standard addition were achieved between 97.5% and 103.3% by using samples (at the level of ng/mL) with 100-fold dilution, which represented the feasibility of this method for low concentrated and complex samples.

#### 4.3.2. Electrochemical Biosensor/Sensor

**Figure 6:**
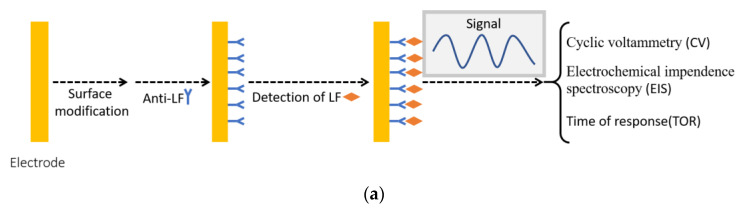

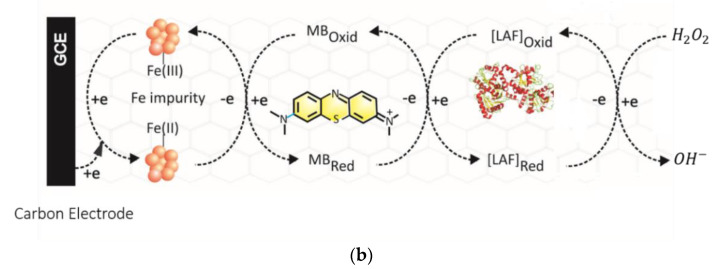
Electrochemical biosensor for the detection of lactoferrin (**a**) affinity sensor with anti-lactoferrin modified on the surface of electrode; (**b**) nonaffinity electrochemical sensor, detecting changes of potential in redox reaction between lactoferrin and hydrogen peroxide (reprinted with permission) [116].

It has been widely posited that electrochemical biosensors work as a platform which could convert biological and chemical changes to electrical signal which could be detected by processor. Typically, such a system consists of a detection target (antibodies, DNA or RNA, and other biomolecules), transducer and signal processor. An electrical signal could be produced as the result of the selective interaction between the target and analyte. Then, it is transmitted via electrode to signal processor, which is responsible for the data amplification and separation. This makes this system acquire high selectivity, high accuracy and low detection limit. There are mainly two different types, namely affinity sensors with anti-LF on the surface of electrode (Figure 6a) and nonaffinity sensors by detecting potential changes in redox reaction regarding LF (Figure 6b).

Electrochemical immunosensors with anti-LF tend to have high specificity to LF and a very low detection limit. Liao’s group [117] successfully developed an anti-LF modified gold electrode sensor with a detection range of 0.01–1000 ng/mL and detection limit of 4.9 pg/mL, as well as shelf life of a month. Although the result with such high sensitivity was innovative and surprising, this process is still largely dependent on an expensive electrochemical system with high resolution, and cyclic voltammetry still needed to be scanned 20 times to achieve the reliable result. Similarly, another immunosensor with multilayer structure was developed by only using a low-cost microcontroller rather than electrochemical station [118]. Such a sensor, having graphene nanoplatelet and polymer complex deposited on the gold electrode and anti-LF on the surface, succeeded in detecting LF in the range of 1 to 10 mg/mL. During the detection process, this system only needs to record time of response to detect LF rather than do repeated signal scanning. This supports the potential application of electrochemical immunosensors. However, the limited shelf life of this affinity sensor may contribute to the relatively high cost. Nonaffinity electrochemical sensor without biomolecules (enzymes, aptamer and antibody) may avoid the shelf-life problem, but the detection process is relatively complicated and lacking selectivity. A multiwalled carbon nanotube modified glassy carbon electrode with the immobilization of methylene blue [116] is reported to have specification detection of LF, and the intensity of signal is proportional to concentration of LF.

#### 4.3.3. Surface Plasmon Resonance (SPR) Sensor

Surface plasmon resonance, including TSPR (transmission surface plasmon resonance), SPRI (surface plasmon resonance imaging), LSPR (localized surface plasmon resonance), and FOSPR (fiber-optic surface plasmon resonance), has been widely used in the quantitive measurement of chemicals, biomolecules and interaction between antibody and proteins [119].

**Figure 7:**
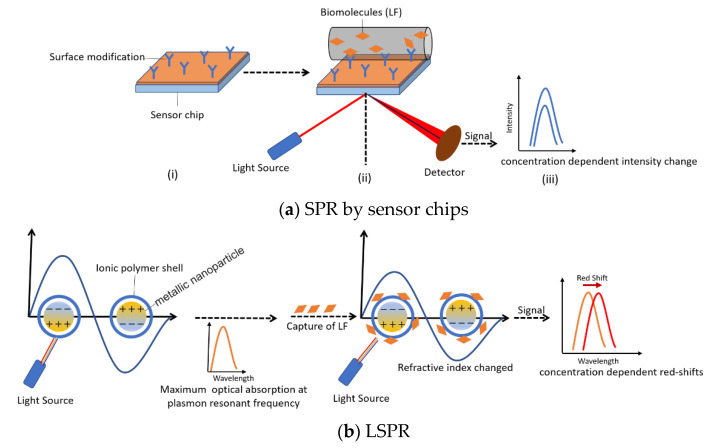
Lactoferrin detection by surface plasmon resonance (SPR) sensor. (**a**) SPR on sensor chips (i. Surface modification on sensor chips or gold nanoparticles; ii. The capture of lactoferrin on the surface was tested by laser beam; iii. Concentration dependent intensity change or red shift was recorded); (**b**) localized surface plasmon resonance (LSPR, the slight change of the refractive index of gold nano-shell after capture of lactoferrin results in the red shift of resonant frequency).

A label-free immunoassay based on SPR was developed by Indyk and Filonzi, which could detect LF in bovine milk with a detection range of 0–1000 ng/mL. The surface of a sensor chip was coated with anti-bovine LF antibody for directly immobilizing LF and the intensity of response reflects the levels of LF [120] (Figure 7a). Similarly, Tomassetti’s research group determined the concentration of LF in cow and goat milk samples by using an immunosensor based on SPR with anti-LF solution directly deposited on the sensor chips. Results of the detection by SPR immunosensor, operated in both batch and flow modes, compared by using classical and screen-printed immunosensors, confirmed that the SPR device can get a lower detection limit of around 10^−8^ M and a measurement time reduced to half [121]. Furthermore, the antibodies for other proteins could also be immobilized on the different channels of sensor chips for simultaneous detection of various whey proteins [122]. Anti-bovine LF as well as four other antibodies were modified on the chips. The results showed high accuracy and selectivity between 0 and 100 ng/mL for all components. However, if the concentration differences among analytes were large, several dilutions with different buffers were required in this method to obtain the proper results. Despite the fact that application of the antibody–antigen interaction could be applied for LF detection by the SPR mechanism, other interactions, such as electrostatic interaction between biomolecules, could also have similar application. An ionic polymer poly(N-isopropylacrylamide-co-methacrylic acid) (PNM) was coated on the surface of silica gold nano-shells for binding proteins which have high isoelectric points, such as LF and lysozyme [123]. Upon the binding of LF and the external polymer, the peaks of LSPR would have red shift, which was concentration dependent and showed good linearity in the range of 0–96 μg/mL (Figure 7b). In terms of LSPR, the size and its distribution of gold nano-shells would largely affect the plasma resonant frequency of itself. This means slight changes in the preparation process or the agglomeration of nanoparticles as time goes on would contribute to the varied adsorption spectrum. Therefore, the standard curves and calibration are required each time before the direct detection of LF.

Table 1 provides the comparison of different detection methods to determine the concentration of LF. Electrophoresis and chromatography techniques are direct quantification methods of LF with small amount of sample and have shown good performance and reproducibility. However, distinguishing LF from the complex samples is still a problem in those fields, especially for those containing proteins with similar pI. Meanwhile, the high price of instruments and the requirement for trained operators are their limitations in the point-of-care testing. ELISA has high accuracy but is considered as a laborious and time-consuming process. RID is one of the simplest methods but with low sensitivity. Both are based on the immunoreaction between LF and its antibody for the detection with high selectivity but the expensive reagents make it difficult for them to be applied in developing daily applications.

The past decade has witnessed substantial progress on the study of various sensors and their applications used for the quantification of biomolecules with high sensitivity and selectivity, and most of them do not require a pre-separation process. Electrochemical sensors have the potential to develop point-of-care devices of LF due to their integrability with existing modules, reliability, accuracy, low LOD and repeatability. Fluorescence-based biosensor is also one of the prospective methods which can be commercialized, and has various advantages, such as the simplicity in operation, ease to observe and low dependence on instruments. The development of the SPR sensor of LF makes the real-time analysis of LF with high accuracy possible. However, an order of magnitude improvement in the LOD of electrochemical sensors can only be achieved with the existence of high-resolution a working station and repeated signal scanning. The affinity electrochemical sensor typically has limited shelf life, which creates a gap for commercialization. In terms of the nonaffinity sensor, it is based on other mechanisms such as the redox reaction. There is no need to consider the shelf life, but it is relatively complicated and lacks selectivity. Fluorescence-based biosensor has difficulties in the control of the quality and uniformity at different batches, which may generate fake and noisy signals, thus affecting the results, while SPR sensors are highly dependent on instruments and the cost of testing is relatively high.

## 5. Conclusions

Lactoferrin, with many biofunctions, has been considered a biomarker for different diseases, e.g., IBD, AD and DED. This review presented methods for the detection of LF, including electrophoresis, chromatography, spectrophotometry, ELISA, RID and sensors (including electrochemical sensor, fluorescence-based biosensor and SPR sensor). Although these methods have been investigated to detect lactoferrin, none of them can obtain all requirements (portability, repeatability, low cost and LOD, high efficiency, accuracy, selectivity and sensitivity). ELISA and RID need costly reagents. The accuracy of RP-HPLC, electrophoresis, some electrochemical sensors and SPR sensors is highly dependent on expensive instruments. The repeatability of sensors can be easily affected by environmental factors. In summary, there is a need to develop a quick and cost-effective LF detection system, and this review paper could make some contributions to the development of novel and advanced detection methods and devices.

## Figures and Tables

**Table 1:** Comparison of different methods for detecting lactoferrin.

	Method	Advantages	Disadvantages
Instrumental analysis	CE	Small amount of sample; High accuracy;	Hard to separate and distinguish LF in complex samples;
	RP-HPLC		
Immune sensor	ELISA	High selectivity; High accuracy; Low limit of detection (LOD) (~3 ng/mL); High through detection;	Expensive reagents; Laborious process; Time-consuming process;
	RID	Not require any instruments; High specificity;	Large systematic error;
Sensor	Electrochemical sensor	High sensitivity; Potential of commercialization;	Limited shelf life (affinity sensor); Relying on working station(high accuracy);
	Fluorescence-based sensor	Operation simplicity; Visualization; Low cost; High selectivity and sensitivity; High accuracy; High through detection;	Quality control; Easy to be affected by environmental factors; Easy to generate fake and noise signal; Laborious preparation process;
	SPR sensor	Real-time analysis; High accuracy (typically 0–1000 ng/mL); Simultaneous detection of various proteins;	Size dependent adsorption spectrum (LSPR); Relatively high cost;
